# The role of expertise in the aesthetic evaluation of mathematical equations

**DOI:** 10.1007/s00426-021-01592-5

**Published:** 2021-09-08

**Authors:** Gregor U. Hayn-Leichsenring, Oshin Vartanian, Anjan Chatterjee

**Affiliations:** 1 Institute for Anatomy I, University Hospital Jena, Jena, Germany; 2 Department of Psychology, University of Toronto, Toronto, ON Canada; 3 Penn Center for Neuroaesthetics, University of Pennsylvania, Philadelphia, PA USA

## Abstract

There is a notion that mathematical equations can be considered aesthetic objects. However, whereas some aesthetic experiences are triggered primarily by the sensory properties of objects, for mathematical equations aesthetic judgments extend beyond their sensory qualities and are also informed by semantics and knowledge. Therefore, to the extent that expertise in mathematics represents the accumulation of domain knowledge, it should influence aesthetic judgments of equations. In a between-groups study design involving university students who majored in mathematics (i.e., experts) or not (i.e., laypeople), we found support for the hypothesis that mathematics majors exhibit more agreement in their aesthetic judgments of equations—reflecting a greater degree of shared variance driven by formal training in the domain. Furthermore, their judgments were driven more strongly by familiarity and meaning than was the case for laypeople. These results suggest that expertise via advanced training in mathematics alters (and sharpens) aesthetic judgments of mathematical equations.

*If one is working from the point of view of getting beauty in one’s equation, and if one has really sound insights, one is on a sure line of progress. Paul Dirac*.

## Introduction

In everyday life, we judge many things based on their aesthetic appeal. Most of these entities are typically objects with sensorial properties like faces, landscapes, cars, paintings, and so on. Furthermore, it is known that we enjoy sensory experiences associated with auditory (e.g., music), tactile (e.g., fur), and olfactory (e.g., scent of a rose or wine) stimuli (Diessner et al., 2019). In addition to these sensory-laden objects, entities that are deemed primarily conceptual in nature can also have aesthetic qualities. One such entity that has historically been the focus of considerable discussion involves mathematical concepts/ideas (Johnson & Steinerberger, 2019), and stimuli that represent them (e.g., equations). For example, the Hungarian mathematician Paul Erdős imagined a book created by God. This (hypothetical) book would include only the most elegant, most surprising and, therefore, most aesthetically appealing proofs for any mathematical proposition. The highest accolade from Erdős for a particular proof was the sentence: “That’s from the book!” (Aigner & Ziegler, 2013). For Erdős, such a book had no tolerance for ugly mathematics (i.e., very complex and counterintuitive mathematical proofs). In essence, when an equation is perceived to be aesthetic, it might give the impression that it reflects truth more so than if it were perceived to be aesthetically displeasing. This belief is shared by other philosophers, mathematicians, and physicists, e.g., Bertrand Russell (Russell, 1917) and Hermann Weyl (Dyson, 1956), suggesting that the best mathematical proofs or concepts have high aesthetic value.

This view, one that conflates beauty and truth in mathematics, is not shared universally (Hossenfelder, 2018). Hossenfelder argued that the search for beauty in mathematics has prevented us from making major breakthroughs, because complex and “ugly” proofs are judged to be inferior. Even more importantly, many beautiful mathematical theories like supersymmetry and grand unification remain untestable, and can therefore interfere with scientific progress. Indeed, it has been shown that mathematicians do not necessarily relate the concept of beauty with that of simplicity (Inglis & Aberdein, 2015). Given that there is no universal agreement about the value of aesthetics in mathematics, this topic is worthy of further investigation. Not only can such an investigation inform us about the contribution of aesthetic factors to mathematics, but at a more general level, it can increase our understanding of factors that drive preference for objects beyond sensory qualities exclusively.

Our focus on the aesthetic experience of mathematical equations is motivated by two different lines of research. First, strikingly, mathematicians from different cultures attach aesthetic value to similar mathematical concepts (Emmer, 2005). This agreement suggests that there is some degree of cultural invariance in factors that drive the perception of beauty in mathematics. Second, beautiful mathematical equations can evoke activity within the same brain areas as other beautiful entities, such as music and visual art (Ishizu & Zeki, 2011; Zeki et al., 2014). Specifically, visual (Kawabata & Zeki, 2004), musical (Blood et al., 1999), moral (Tsukiura & Cabeza, 2011) and mathematical (Zeki et al., 2014) beauty judgments all correlate with activity in the medial orbito-frontal cortex—a structure associated strongly with reward. Consistent with the “common currency hypothesis” (Levy & Glimcher, 2012), this correlation suggests that this region of the brain computes and represents the pleasure derived from beauty across a wide set of entities, including mathematics.

At this point, we might wonder what drives aesthetic judgments of mathematical equations. It seems reasonable to assume that, at least in part, the aesthetic experience of mathematics depends on learning (Zeki et al., 2014, 2018). Presumably, people not well versed in the language of mathematics would be unable to have a full-fledged aesthetic experience involving mathematical concept. For example, many mathematicians regard Euler’s identity (e^*iπ*^ + 1 = 0) as one of the most beautiful equations (Chatterjee, 2015), a claim beyond the grasp of most laypeople who might not be able to observe the deep conceptual underpinnings of the equation, and thus its meaning. Consistent with this idea, in an experimental study with mathematicians (i.e., domain experts) as participants, Zeki and colleagues (2014) found that beauty ratings correlated negatively with standard deviations in judgments of equations, i.e., there was a greater consensus on beautiful mathematical equations than on not beautiful equations. The authors interpreted this finding to mean that a sense for the beauty of equations is shared and universal, but that it can only be perceived when people *understand* those equations (or have learned the “language” of mathematics).

Here, we propose a different, but related, framing for aesthetic experiences involving mathematics. We argue that laypeople (i.e., university undergraduates with no advanced training in mathematics) are also capable of performing aesthetic judgments in the domain of mathematics, although to a less degree than domain experts. This is because even laypeople will have had some rudimentary exposure to mathematics in primary and secondary school, giving them a basic understanding of numbers and symbols systems. Indeed, it has been shown that laypeople can experience mathematics aesthetically (Johnson & Steinerberger, 2019). However, they will lack the depth and breadth of knowledge associated with advanced training in mathematics, making them relatively less capable of deriving meaning from equations to the same extent that experts and quasi-experts in mathematics will be able to do. Indeed, within the model of the “aesthetic triad”, aesthetic experiences are considered to emerge from the interaction of three neural systems: knowledge meaning, sensory motor, and emotion valuation (Chatterjee & Vartanian, 2014, 2016). Aesthetic experiences can be triggered by sensory information, as is the case for faces, landscapes, and human artifacts such as cars, vases, architecture, and artworks. When compared with natural objects, such as faces and landscapes, human artifacts allow for more variability in aesthetic judgment. For example, Vessel and colleagues (2018) demonstrated that people agree more often on beauty in faces and scenes than in artworks and architecture. They concluded that “artifacts of human culture, which lack uniform behavioral relevance for most individuals, require more individual aesthetic sensibilities that reflect varying experiences and different sources of information” (p. 121). Presumably, this pattern would also apply to mathematical formulae, given that they represent a highly specialized domain of human artifacts that draw heavily on the knowledge base of the observer. In the context of the aesthetic triad, the knowledge-meaning system would likely be a stronger contributor to the computation of aesthetic judgment for mathematical equations among people with expertise in mathematics as compared to those without such domain expertise.

Here it is important to note that we are not making the argument that mathematical equations are devoid of sensory qualities. On the contrary, the visual information inherent in symbol systems can evoke sensory responses in the brain. However, we argue that mathematical concepts and ideas (e.g., relationships between numbers and symbols) represent qualities that extend beyond the sensory domain, and impact the knowledge-meaning system in the brain (see Chatterjee & Vartanian, 2014, 2016). Specifically, mathematical concepts reflect postulated relationships among elements, such that knowledge and meaning are the primary conduits into the computation of their aesthetic value. Following this line of reasoning, students with advanced training in mathematics should agree more in their aesthetic judgments of equations than laypeople. This is because whereas laypeople might primarily rely on sensory information represented by equations (e.g., shapes and relative size of numbers and symbols), people with domain expertise in mathematics can apply *shared* knowledge of these equations in a way that is not accessible to laypeople for generating aesthetic evaluations.

To test this prediction, we conducted an experiment on aesthetic judgments of mathematical equations, with expertise as a between-subjects factor. We tested three hypotheses: First, we hypothesized that there would be more agreement among students of mathematics (i.e., experts) than laypeople when making aesthetic judgments of mathematical equation (*Hypothesis 1*). Testing this hypothesis would inform us about the effects of expertise on aesthetic judgments in the domain of mathematics (Brinkmann et al., 2014; Silvia, 2013). We reasoned that the greater degree of agreement in aesthetic judgments among students of mathematics than laypeople would reflect a greater degree of shared variance driven by advanced training in the domain in the former group.

Second, we hypothesized that certain features of the stimuli (i.e., mathematical equations) might drive aesthetic judgments to a different extent in students of mathematics than in laypeople (*Hypothesis 2*). Testing this hypothesis would indicate whether relevant knowledge is consciously accessible and brought to bear by mathematics experts more so for making aesthetic judgments as compared to laypeople. Regarding Hypothesis 2, three factors that were of particular interest to us consisted of familiarity (*Hypothesis 2a*), meaning (*Hypothesis 2b*), and complexity (*Hypothesis 2c*). Specifically, we hypothesized that due to their advanced training in mathematics, the aesthetic judgments of experts would be more likely to be driven by the extent to which the equations appeared familiar (because of greater likelihood of past exposure), meaningful (because of their ability to understand the relationships that the equations denote), and complex (because of their ability to process more elements in the stimuli). Indeed, there is a substantial literature in empirical aesthetics demonstrating that experts but not nonexperts prefer more complex art, perhaps due to their ability to process a greater frequency and diversity of elements in the stimuli (see Van Geert & Wagemans, 2020).

We tested these two hypotheses with equations as stimuli. Equations can be presented as discrete stimuli and are easier to process than larger mathematical proofs. In addition, although they are presented visually, equations represent mathematical concepts that might or might not be intelligible to the perceiver as a function of background knowledge and/or relevant expertise. Therefore, equations are suitable stimuli for empirical research on aesthetics in the domain of mathematics.

## Method

### Participants

This study was approved by the ethics committee of the University of Maine, and all participants stated their willingness to participate in the study by signing a consent form. Forty participants participated in the study. Twenty of those participants were people with a mathematics degrees or university students of mathematics (experts; mean age = 20.9 years, 5 female), whereas the other twenty participants were university-level students who did not have advanced training in mathematics (laypeople; mean age = 23.5 years, 12 female). There was a greater proportion of males than females among experts than laypeople, Chi-square, *X*^2^ (1, *N* = 40) = 5.01, *p* = 0.025. There was no difference in age between the two groups (*t*[38] =  − 1,698, *p* = n.s.).

### Stimuli

We used 64 index cards with mathematical equations printed on each card (see Supplemental Table 4). Mathematical equations were selected with the help of a mathematics professor at the University of Maine to ensure that they represented a wide range of subfields within mathematics, as well as degrees of mathematical beauty as judged by an expert in the field. Index cards had a size of 7.5 × 12.5 cm.

### Procedures

First, we asked participants to perform the Visual Aesthetic Sensitivity Test (VAST, Gear, 1986) in order to assess general aesthetic sensitivity. For the VAST participants were presented with 50 pairs of pictures with quite similar paintings. One of the two paintings was considered superior in design (e.g., more harmonious, better balanced, better ordered, etc.). Participants were asked to determine which picture was better designed. The VAST was administered to ensure that the experts and laypeople did not differ in their general aesthetic sensitivity as measured by this instrument.

In the main part of the study, we asked participants to sort mathematical equations using the Q-Sort technique (QST, Previte et al., 2007). In QST, participants are asked to sort a number of objects—in this case cards with mathematical equations—by placing a specific number of statements in a predetermined distribution pattern (here: normal distribution) along a spectrum of ranking categories (here: “least aesthetic” to “most aesthetic”). When compared with a simple rating task, the QST has the advantage that every participant is forced into the same categorization pattern, making the results (i.e., relative sorting pattern) comparable between groups of participants. More importantly, it is well-suited for assessing differences in the degree of agreement in ratings between experts and laypeople, which represented the main aim of the current study.

In this particular experiment, participants performed two rounds of sorting. In the first round, we asked them to place each of the shuffled cards on one of three piles (“unaesthetic”, “neither unaesthetic nor aesthetic”, “aesthetic”) without restricting the number of cards per pile. In the second round, we asked the participants to redistribute the cards from those piles into nine piles ranging from “extremely unaesthetic” (pile 1) to “extremely aesthetic” (pile 9). A number of cards in each of the nine piles were predetermined by the experimenter (3, 5, 7, 10, 14, 10, 7, 5, 3, respectively) to obtain a normally distributed pattern. The participants received no further instructions on the use of the term “aesthetic,” and they were given a maximum time of one hour to complete both rounds of the main experiment. The sorting occurred in two steps to make it easier for the participants to complete a coarser sorting first, before producing a more fine-grained sorting pattern. Advance piloting at the University of Maine had shown that this two-step procedure helped participants in completing the sorting task, and that the amount of time (i.e., one hour) was sufficient for sorting the 64 cards in accordance with these instructions. All analyses were performed on the second sorting of the equations.

After completing the experiment, we asked the participants to indicate the equations with which they were familiar prior to this study. The experimenter wrote down the answers. Additionally, we asked them to fill out a form stating their criteria for classification, which included the following options: “simplicity”, “balance”, “complexity”, “symmetry”, “form”, “composition”, “meaning”, and “other”. Specifically, they were asked to check a box next to any criterion that was taken into consideration during the Q-sort process. We opted for this approach for two reasons. First, providing some relevant options based on input from the mathematics professor at the University of Maine as well as our reading of the literature offered our participants a framework for considering the task. Importantly, including the “other” option enabled them to list other factors beyond what the experimenters considered relevant. However, no participant chose this option, and as such we omitted it from the analysis. Second, we opted for a binary approach (checked/not checked) in relation to the entire sorting exercise to get a sense of which factors were considered generally relevant when assessing the aesthetics of mathematical equations.

## Results

We found no difference in the VAST results between experts and laypeople (independent samples student’s *t* test: *median*_*experts*_ = 38, *SD*_*experts*_ = 3.4; *median*_*laypeople*_ = 39, *SD*_*laypeople*_ = 4.1; Cohen’s *d* =  − 0.180, *p* = 0.430). This suggests that the two groups did not differ in their general ability to make aesthetic judgments based on the visual stimuli.

**Fig. 1:**
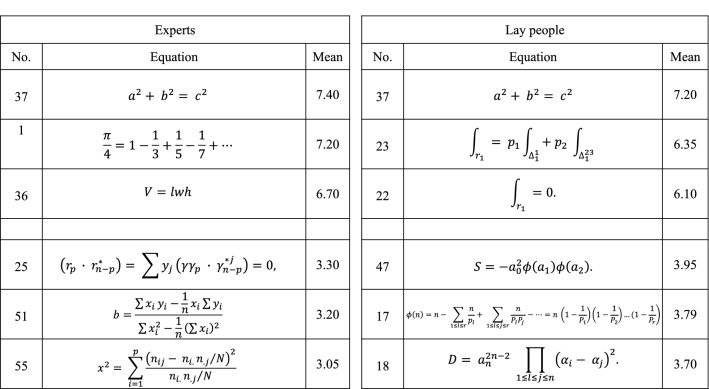
Equations categorized as most and least aesthetic by experts and laypeople

See Fig. 1 for some of the best- and worst-rated equations by experts and laypeople. We had predicted that there would be more agreement among students of mathematics than laypeople when making aesthetic judgments of mathematical equation (*Hypothesis 1*). Consistent with this prediction, for Q-sort task, Cronbach’s *α* was higher for experts (*α* = 0.84) than for laypeople (*α* = 0.60). A Chi-squared test showed that experts agreed with one other in their classification of the equations more so than did laypeople, *X*^2^ (1, *n* = 64) = 11.497, *p* < 0.001.

Our next focus involved exploring whether familiarity and meaning were relevant to the participants for categorizing equations in terms of their aesthetic appeal (*Hypotheses 2a and 2b*). Unsurprisingly, on average, experts were more familiar with the equations than were laypeople (independent samples student’s *t* test: *median*_*experts*_ = 21, *SD*_*experts*_ = 9.9; *median*_*laypeople*_ = 12, *SD*_*laypeople*_ = 10.8; Cohen’s *d* = 0.588, *p* < 0.05). As for the aesthetics justification, experts stated more often than laypeople that their classification relied on meaning (Chi-squared test: *X*^2^ [1, *N* = 40] = 5.867, *p* < 0.05). See Table 1 for the other categories.

**Table 1:** Factors that were highlighted as drivers of aesthetic evaluation of mathematical equations—broken down by condition (i.e., experts vs. laypeople)

	Familiarity (median number of equations)	Simplicity	Balance	Complexity	Symmetry	Form	Composition	Meaning
Experts	**21**	17	9	12	8	11	10	**14**
Laypeople	**12**	16	5	9	8	11	7	**7**

Bold values indicate *p* < 0.05. Displayed are the median number of familiar equations (out of 64) and the number of participants (out of 20 for each group) that based their aesthetic categorization on certain factors

Following this same line of exploration, we then examined the extent to which the factors that were highlighted as drivers of aesthetic evaluation of mathematical equations were correlated with performance on the Q-sort task. In the analysis of the experimental data, we firstly looked for familiarity with the equations. With 9 piles of cards (pile 1 “extremely unaesthetic” to pile 9 “extremely aesthetic”), an average assignment would be 5. For experts, we found a median value of 6.00 (*SD* = 0.73) for familiar equations, whereas for laypeople we found a median value of 5.65 (*SD* = 1.01) for familiar equations. Paired-sampled Student’s *t* test showed that both groups had significantly higher assignments of familiar equations as compared to unfamiliar equations (experts: *t*[18] =  − 7.696, *p* < 0.001; laypeople: *t*[18] = -2.864, *p* = 0.01). Independent samples Student’s *t* test showed no difference between the groups (*t*[36] = 1.399, *p* = 0.17), suggesting that the two groups preferred familiar equations over unfamiliar equations to a similar extent.

In the next step, we counted how many experts and how many laypeople were familiar with each *specific* Eq. (1–64). The number of experts (and laypeople, respectively) that were familiar with each single equation was then correlated with average classification by experts and laypeople, respectively. We found a significantly higher correlation for average experts’ aesthetic categorization with number of experts familiar with the respective equations (*ρ*_*experts*_ = 0.793, *p* < 0.001), compared to laypeoples’ average aesthetic categorization with number of laypeople familiar with the respective equations (*ρ*_*laypeople*_ = 0.564, *p* < 0.001, Fisher’s *z*: *p* < 0.01) (see Table 2). This means that experts preferred equations that they (as a group) were more familiar with to a higher degree than laypeople preferred equations that they (as a group) were more familiar with. In addition, we calculated the difference in number of experts familiar with each single equation and number of laypeople familiar with the same equation and, again, correlated this value with aesthetic classification by experts and laypeople (*ρ*_*experts*_ = 0.444, *p* < 0.001; *ρ*_*laypeople*_ = 0.109, *p* = 0.392, Fisher’s *z*: *p* < 0.001) (see Table 2). This means that experts preferred equations that were more familiar to them (as a group)—whereas no such effect was seen for laypeople.

**Table 2:** Matrix for Spearman’s *ρ* correlations between number of participants familiar with the equation and average categorization

	Average (experts)	Average (laypeople)	Fisher’s *z*
Number of participants familiar with this equation	**0.758**	**0.575**	*p* = 0.032
Number of experts familiar with this equation	**0.793**	**0.509**	*p* = 0.002
Number of laypeople familiar with this equation	**0.590**	**0.564**	*p* = n.s
Difference between number of experts and number of laypeople familiar with this equation	**0.444**	0.109	*p* < 0.001

Bold values indicate *p* < 0.001. Fisher’s *z* indicates significant differences in size between the experts’ and laypeoples’ correlation. There was a significant difference (Fisher’s *z*: *p* = 0.007) between the correlations “number of experts familiar with this equation—average (experts)” (*r* = 0.793) and “number of laypeople familiar with this equation—average (laypeople)” (*r* = 0.564)

Next, we analyzed classifications in relation to the complexity of the equations (*Hypothesis 2c*). As a measure for complexity, we counted the number of elements within each equation (count of numbers, count of Latin letters, count of Greek letters, count of mathematical signs and count of all elements). We found a negative correlation of aesthetic classifications with count of all elements for experts (Spearman’s *ρ* =  − 0.540, *p* < 0.001), but no correlation for laypeople (*ρ* =  − 0.133; *p* = 0.097) (see Table 3). Using Fisher’s *z*, we confirmed that the difference between the two correlations was statistically significant (*p* < 0.001). This observation means that experts preferred equations with fewer elements, while the count of elements did not affect the aesthetic preference for laypeople.

**Table 3:** Spearman’s *ρ* correlation between complexity (number of elements in the equations) and average categorization

	Experts	Laypeople	Fisher’s *z*
Numbers	− 0.197	− 0.071	*p* = n.s
Latin letters	− **0.485**	− 0.009	*p* < 0.001
Greek letters	0.017	0.020	*p* = n.s
Signs	− **0.507**	− 0.244	*p* = n.s
Sum of all elements	− **0.540**	− 0.133	*p* < 0.001

The total number of elements over all 64 equations is 1486 (numbers: 203; Latin letters: 563; Greek letters: 36; signs: 684). Bold values indicate *p* < 0.001. Fisher’s *z* indicates significant differences in size between the experts’ and laypeoples’ correlation

## Discussion

We conducted this study to test two overarching hypotheses. First, we hypothesized that students of mathematics would agree more than laypeople when making aesthetic judgments of mathematical equations (*Hypothesis 1*). We found support for this hypothesis. We believe that the higher degree of agreement among experts reflects a greater degree of shared variance, driven by advanced training in the relevant domain.

Second, we tested the hypothesis that certain features of the stimuli (i.e., mathematical equations) might drive aesthetic judgments to a different extent in students of mathematics than laypeople (*Hypothesis 2*). Our first focus was on familiarity (*Hypothesis 2a*). Our results demonstrated that although in general students of mathematics were more familiar with the equations than laypeople, familiarity nevertheless mattered to both groups of participants: experts and laypeople preferred those equations that appeared more familiar to them. However, there were also differences between the two groups: when we shifted our focus to degree of familiarity with each *specific* equation, we found that experts preferred equations that they (as a group) were more familiar with to a higher degree than laypeople preferred equations that they (as a group) were more familiar with. Next, when we calculated the difference in number of experts familiar with each single equation and number of laypeople familiar with the same equation, we found that experts preferred equations that were more familiar to them (as a group)—whereas no such effect was seen for laypeople. These findings suggest that overall, there are both similarities and differences in the ways in which familiarity exerts an influence on aesthetic judgment of among experts and laypeople, although there is converging evidence that experts as a group are more influenced by familiarity than are laypeople.

When we shifted our attention to meaning (*Hypothesis 2b*), our results demonstrated that understanding the meaning of an equation is an important factor that contributes to its aesthetic appeal, especially among experts. This indicates that relevant domain knowledge is consciously accessible and brought to bear by mathematics experts for making aesthetics judgments. Finally, experts but not laypeople preferred lesser complex equations (i.e., those that contain fewer elements) (*Hypothesis 2c*). This finding contradicts our prediction that experts would prefer more complex equations. However, it is in line with the historical association outlined between mathematical beauty and simplicity. As described by Paul Erdős, elegance—and therefore, simplicity—is a key factor for a beautiful mathematical proposition (Aigner & Ziegler, 2013). Interestingly, when mathematicians were asked to describe mathematical proofs, no correlation was found between the terms beauty and simplicity (Inglis & Aberdein, 2015). Clearly, more research is needed to elucidate the relationship between beauty and complexity/simplicity in the domain of mathematics.

Combining our results, we infer that among mathematics students, preferences are more likely to be a function of not only the sensorial qualities of the equations, but also the degree of familiarity and the meaning associated with the formulae. In contrast, among laypeople, preferences might be a function of the more sensorial features of the equations, such as the shape and/or relative sizes of the numbers and the symbols within equations. These differences are consistent with previous findings in empirical aesthetics in terms of how domain experts vs. novices evaluate other visual stimuli, such as artworks. For example, Hekkert and van Wieringen (1996) presented persons with and without expertise in the visual arts with original and black- and-white versions of the same postimpressionistic paintings, and showed that in the case of nonexperts the removal of color had a significantly stronger negative impact on aesthetic preference than was the case among experts. This suggested that nonexperts largely base their preference on superficial features of artworks (e.g., color), whereas preference among experts is driven by the structural properties of the artwork (e.g., geometric relationship of the objects within the work), which remain unchanged despite the removal of colour information. We believe that something similar might be at play here, such that mathematics students are able to base their aesthetic preferences on shared meaning and knowledge, whereas laypeople base their aesthetic preference relatively more on more sensorial and superficial features of the equations. Mathematics students are familiar with the structure and content of the equations in general, as well as their meanings based on the advanced training. This knowledge base can in turn contribute to a well-developed aesthetic taste for equations, consistent with findings that expertise derived from advanced training exerts a robust and reliable effect on judgment (Ericsson et al., 2018; Tinio et al., 2014).

Our findings are also in line with the prediction derived from the “aesthetic triad” model (Chatterjee & Vartanian, 2014, 2016). Specifically, expertise in mathematics modifies the aesthetic perception of the respective stimuli in that domain. The fact that the groups did not vary in their general aesthetic sensitivity as measured by the VAST suggests that the two groups did not differ on some general factor of taste, if such a factor even exists. Any difference in group performance is more likely to be rendered by differences in relevant domain knowledge. To be more precise: mathematics students varied to a far less degree in their categorization of equations as compared to laypeople because the knowledge-meaning system is more important for the aesthetic appeal of mathematical equations for that group, the content of which is more likely to be shared among domain experts. Although equations are visual stimuli (i.e., participants visually inspected them in our experiment), in the present context the sensory-motor system would appear to be relatively less important than it is in relation to other stimuli (e.g., faces, landscapes, and artworks). Having said this, we acknowledge that among mathematics students both the knowledge meaning as well as sensory sources of information contribute to aesthetic evaluation, although perhaps to different extents.

Our findings are also in line with the model proposed by Redies (2015), according to which perceptual and cognitive processing interact with each other in leading to aesthetic appreciation. For mathematical equations, perceptual processing is relatively less relevant. Subsequently, aesthetic experience is altered by knowledge, because in this case the cognitive processing is of utmost importance for the aesthetic appreciation of mathematical equations. We speculate that equations that are experienced as aesthetically pleasing might be processed more fluently (Reber et al., 1998). Here, of course, fluency would be driven by meaning and the associated understanding of concepts and their relationships, rather than their sensual qualities exclusively. This idea is also in line with the observed differences between experts and laypeople, such that the shared knowledge among experts would be expected to contribute to more homogeneity in how equations are processed—as we have observed here.

In terms of future work, we hope that the findings presented here will encourage researchers in empirical aesthetics to broaden the scope of their investigations to explore the impact of aesthetics in domains beyond (visual) art, music, and dance, among others. Specifically, by embracing what Martindale (1984) referred to as “cognitive hedonics,” there is an opportunity to study aesthetic pleasure associated with *ideas*—a largely neglected topic in empirical aesthetics. In this sense, equations represent one stimulus category embodying ideas in mathematics, but others certainly exist (e.g., symbol systems in language). This endeavour will deepen our understanding of the contribution of the knowledge-meaning system to the emergence of aesthetic experiences (see Chatterjee & Vartanian, 2014, 2016).

### Limitations

Our study had several limitations. First, we chose the Q-sort method for data collection because our focus was primarily on whether experts would exhibit more agreement in their ratings of mathematical equations than laypeople. The Q-sort method is well suited for tackling this question because it examines the relative (rather than independent) preference levels assigned to a set of equations. However, the Q-sorts method does not allow one to analyze whether experts or laypeople, *on average*, vary in their overall evaluation of mathematical equations—a question that is important to study in its own right. Second, we did not have an independent measure of the mathematical skills of the laypeople, aside from the fact that they lacked advanced training in mathematics. Our assumption was that they completed classes in mathematics in high school, and that *relatively speaking*, they were less knowledgeable in mathematics than mathematics majors. Future studies should collect independent measures of mathematics knowledge and skills. Third, our design did not enable us to conduct an in-depth analysis of whether mathematics students were indeed more aware of *why* they preferred certain equations over others, because we did not collect preference ratings for each equation independently. Fourth, we did not measure how important each criterion (e.g., simplicity) was relative to others for making aesthetic judgments. Collecting such data would enable one to test specific hypotheses with a higher degree of granularity than was presently possible. We hope to address these questions in further research.

### Summary

We provided evidence to show that mathematics students agreed more in their ratings of the aesthetics of mathematical equations than did laypeople—reflecting what we believe is a greater degree of shared variance driven by advanced training in the relevant domain. We found that mathematics majors reported preferring lesser complex equations. In addition, their judgments were driven more strongly by familiarity and meaning than was the case for laypeople. These results confirm our overall prediction that expertise alters (and sharpens) aesthetic judgments of mathematical equations, and that advanced training in mathematics might alter the impact of certain factors on aesthetic judgment in the domain of mathematics.

## Appendix 1

Table 4.

**Table 4:** All the equations that were used in our experiment

1	$\frac{\pi }{4} = 1 - \frac{1}{3} + \frac{1}{5} - \frac{1}{7} + \ldots$	17	$\phi \left( n \right) = n - \mathop \sum \limits_{1\; \le \;l\; \le \;r} \frac{n}{{p_{l} }} + \mathop \sum \limits_{1\; \le \;l\; \le \;j\; \le \;r} \frac{n}{{P_{l} P_{j } }} - \ldots = n \left( {1 - \frac{1}{{P_{1} }}} \right)\left( {1 - \frac{1}{{P_{2} }}} \right) \ldots \left( {1 - \frac{1}{{P_{r} }}} \right)$
2	${\text{e}}^{i\pi } \; = \;\cos \pi + i\sin \pi = \; - 1 + i \cdot 0 = \; - 1$	18	$D = a_{n}^{2n\; - 2} \mathop \prod \limits_{1\; \le \;l\; \le \;j\; \le \;n} \left( {\alpha_{i} - \alpha_{j} } \right)^{2} .$
3	$s^{2} = \left( {x^{1} - y^{1} } \right)^{2} + \ldots + \left( {x^{n} - y^{n} } \right)^{2}$	19	$\left( {x_{1} \;y_{1} } \right) = \left( {x_{0} + \frac{b}{d}t,\;y_{0} - \frac{a}{d}t} \right),$
4	$\phi = 1 + \frac{1}{{1 \frac{1}{1 + \ldots }}}$	20	$\left( {l,\;m,\;n} \right) = \frac{{\left( {\left| {t_{1}^{2} - t_{2}^{2} - t_{3}^{2} } \right|,2\left| {t_{1} \;t_{2} } \right|,2\left| {t_{1} \;t_{3} } \right|} \right)}}{{t_{1}^{2} + t_{2}^{2} + t_{3}^{2} }}$
5	${}^{N}P_{r} = N \times \left( {N - 1} \right) \times \ldots \left( {N - r + 1} \right)$	21	$f = a\left( {x + \frac{b}{2a}y} \right)^{2} + \frac{{4ac - b^{2} }}{4a}y^{2} .$
6	$$\begin{gathered} \left( {x^{2} y - y^{2} - y^{2} z} \right)dx + \left( {xy^{2} - x^{2} z - z^{2} } \right)dy \\ + \left( {xy^{2} + x^{2} y} \right)dz = 0. \\ \end{gathered}$$	22	$\int_{{r_{1} }} {} = \;0.$
7	$$^{N} C_{r} = \left( {\begin{array}{*{20}c} N \\ r \\ \end{array} } \right) = \frac{{N \times \left( {N - 1} \right) \ldots \left( {N - r + 1} \right)}}{{r \times \left( {r - 1} \right) \ldots 1}}$$	23	$\int_{{r_{1} }} {} = p_{1} \int_{{\Delta_{1}^{1} }} {} + p_{2} \int_{{\Delta_{1}^{23} }} {}$
8	$$\left( {\begin{array}{*{20}c} N \\ r \\ \end{array} } \right) = \frac{N!}{{r! \times \left( {N - r} \right)!}}$$	24	$D = \left| {\left( {\gamma_{p}^{i} \cdot \gamma_{n\; - p}^{*j} } \right)} \right| \ne 0.$
9	$$\left( {\begin{array}{*{20}c} N \\ r \\ \end{array} } \right)\; = \;\left( {\begin{array}{*{20}c} N \\ {N\; - \;r} \\ \end{array} } \right)$$	25	$\left( {r_{p } \cdot r_{n\; - p}^{*} } \right) = \sum y_{j } \left( {\gamma \gamma_{p} \cdot \gamma_{n\; - p}^{*j} } \right) = 0,$
10	$$P\left( r \right) = \left( {\begin{array}{*{20}c} N \\ r \\ \end{array} } \right)p^{r} \left( {1 - p} \right)^{N - r}$$	26	$E_{p} \times E_{q} = - \left( { - E_{p} } \right) \times E_{q} = - E_{p} \times \left( { - E_{q} } \right).$
11	$\mu = 0 \times P\left( 0 \right) + P\left( 1 \right) + 2 \times P\left( 2 \right) \ldots N \times \;P\;(N)$	27	$R_{k} \left( {K \times K^{\prime};\;\Lambda } \right) = \sum R_{S} \left( {K;\;L} \right)R_{k - s} \left( {K^{\prime};\;L^{\prime}} \right).$
12	$\sigma^{2} = \left( {0 - \mu } \right)^{2} P\left( 0 \right) + \left( {1 - \mu } \right)^{2} P\left( 1 \right) + \ldots + \left( {N - \mu } \right)^{2} P\left( N \right).$	28	$\frac{du}{{dx}} + bu^{2} = cx^{2}$
13	$\sigma^{2} = \mathop \sum \limits_{r = 0}^{N} \left( {r - \mu } \right)^{2} P\left( r \right).$	29	$p = p + x\frac{dp}{{dx}} + f\left( p \right)\frac{dp}{{dx}},$
14	$\phi \left( n \right) = n\left( {1 - \frac{1}{{p_{1} }}} \right)\left( {1 - \frac{1}{{p_{2} }}} \right) \ldots \left( {1 - \frac{1}{{p_{r} }}} \right).$	30	$Pdx + Qdy + Rdz = 0$
15	$\mathop {\lim }\limits_{x \to \infty } \pi \left( x \right)\frac{{\log_{e} x}}{x} = 1.$	31	$\frac{dz}{{dx}} = \frac{y}{{\sqrt {\left( {y^{2} - x^{2} } \right)} }}.$
16	$\mathop \sum \limits_{d|n} \phi \left( d \right) = n,$	32	$\frac{dz}{{dx}} = \frac{y}{x + z}.$
33	$\frac{df}{{dx}} + \frac{df}{{da}}\frac{da}{{dx}} + \frac{df}{{db}}\frac{db}{{dx}} = \frac{d\aleph }{{dx}},$	49	$Z = \frac{{\overline{x} - \mu_{n} }}{{\frac{\sigma }{\sqrt n }}}$
34	$\frac{d\phi }{{dy_{1} }}dy_{1} + \frac{d\phi }{{dy_{2} }}dy_{s} = 0$	50	$v = \left\{ {\frac{{\left\{ {\frac{{s_{1}^{2} }}{{n_{1} }} + \frac{{s_{2}^{2} }}{{n_{2} }}} \right\}^{2} }}{{\frac{{s_{1}^{4} }}{{n_{1}^{2} \left( {n_{1} - 1} \right)}} + \frac{{s_{2}^{4} }}{{n_{2}^{2} \left( {n_{2} - 1} \right)}}}}} \right\}$
35	$m\left( {\overline{AB} \cup \overline{CD} } \right) = m\left( {\overline{AB} } \right) + m\left( {\overline{CD} } \right) - m\left( {\overline{AB} \cap \overline{CD} } \right).$	51	$b = \frac{{\sum x_{i} y_{i} - \frac{1}{n}x_{i} \sum y_{i} }}{{\sum x_{i}^{2} - \frac{1}{n}\left( {\sum x_{i} } \right)^{2} }}$
36	$V = lwh$	52	$r = \frac{{\sum \left( {x_{i} - \overline{x}} \right)\left( {y_{i} - \overline{y}} \right)}}{{\left[ {\sum \left( {x_{i} - \overline{x}} \right)^{2} \sum \left( {y_{i} - \overline{y}} \right)^{2} } \right]^{\frac{1}{2}} }}.$
37	$a^{2} + b^{2} = c^{2}$	53	$C = 1 + \frac{1}{{3\left( {K + 1} \right)}}\left\{ {\sum \frac{1}{{\left( {n_{j} - 1} \right)}} - \frac{1}{{\sum \left( {n_{j} - 1} \right)}}} \right\}$
38	$\frac{AB}{{A^{\prime}B^{\prime}}} = \frac{BC}{{B^{\prime}C^{\prime}}} = \frac{CA}{{C^{\prime}A^{\prime}}}$	54	$X^{2} = \mathop \sum \limits_{i = 1}^{K} \frac{{\left( {O_{i} - E_{i} } \right)^{2} }}{{E_{i} }}$
39	$\Delta \left( \frac{a}{b} \right) \approx \frac{b\Delta a - a\Delta b}{{b^{2} }}.$	55	$x^{2} = \mathop \sum \limits_{i = 1}^{p} \frac{{\left( {n_{ij} - n_{i.} n_{.j} /N} \right)^{2} }}{{n_{i.} n_{.j} /N}}$
40	$\left\{ {A,B,C,\left. D \right\}} \right. \cap \left\{ {C,D,\left. E \right\}} \right. = \left\{ {C,\left. D \right\}} \right..$	56	$Z = \frac{{\beta_{2} - \beta_{2} }}{{\sqrt {\sum \left( {\frac{1}{{f_{i} }}} \right)} }}$
41	$\left( {x + \frac{q}{p}} \right)\left( {x^{\prime} + \frac{q}{p}} \right) = \frac{{q^{2} }}{{p^{2} }} - \frac{s}{p}.$	57	$x \wedge y = y \wedge x$
42	$V\left( {AD,BC} \right) = \left( {z_{1} z_{2} ,\;z_{3} z_{4} } \right).$	58	$\left( {x \wedge \left( {y \vee z} \right)} \right) = \left( {x \wedge y} \right) \vee \left( {x \wedge z} \right)$
43	$\frac{AB}{{AC}} \cdot \frac{DC}{{DB}} = \left| k \right|,$	59	$\left( {x \wedge x^{\prime}} \right) = 0$
44	$\left( {x + \sqrt {x^{2} - bc } } \right)\left( {y + \sqrt {y^{2} - ca} } \right)\left( {z + \sqrt {z^{2} - ab} } \right) = abc$	60	$\left( {x \vee 0} \right) = x$
45	$2xyz - ax^{2} - by^{2} - cz^{2} + abc = 0.$	61	$h\left( {\left\{ q \right\}} \right) = h\left( {\bigcap\limits_{q \in I} I \cap \bigcap\limits_{q \notin I} {\left( {I^{\prime}} \right)} } \right)$
46	$\frac{ayz}{{\left( {y - z} \right)^{2} }} = \frac{bzy}{{\left( {z - x} \right)^{2} }} = \frac{cxy}{{\left( {x - y} \right)^{2} }}$	62	$0 \wedge x = \left( {x \wedge x^{\prime}} \right) \wedge x$
47	$S = - \;a_{0}^{2} \phi \left( {a_{1} } \right)\phi \left( {a_{2} } \right).$	63	$$\begin{array}{*{20}c} {T\frac{e}{N}} \\ {var} \\ \end{array} = I_{P} = \begin{array}{*{20}c} {T\frac{e}{N}} \\ {proper} \\ \end{array}$$
48	$\log z = 2\left\{ {\frac{z - 1}{{z + 1}} + \frac{1}{3}\left( {\frac{z - 1}{{z + 1}}} \right)^{2} + \ldots } \right\}$	64	$$\begin{array}{*{20}c} {T_{{\overline{N}}} } \\ {\text{var}} \\ \end{array} = T_{{\overline{N}}} .$$
